# Demographic Vulnerability and Mental Health in Older Adults with Type 2 Diabetes: A Lifespan-Informed Analysis of Diabetes Distress

**DOI:** 10.3390/diseases14090332

**Published:** 2026-09-11

**Authors:** Adriana Gherbon, Deiana Roman, Marioara Nicula-Neagu, Mirela Frandes

**Affiliations:** 1 Department VII Internal Medicine—Diabetes, Nutrition, Metabolic Diseases, and Systemic Rheumatology, “Victor Babeș” University of Medicine and Pharmacy, 300041 Timișoara, Romania; gherbon.adriana@umft.ro; 2 Center of Molecular Research in Nephrology and Vascular Disease, “Victor Babeș” University of Medicine and Pharmacy, 300041 Timișoara, Romania; 3 Diabetes, Nutrition, and Metabolic Diseases, “Pius Brînzeu” Emergency Hospital, 300723 Timișoara, Romania; 4 Second Department of Internal Medicine, “Victor Babeș” University of Medicine and Pharmacy, 300041 Timișoara, Romania; 5 Railway Clinical Hospital, 300173 Timișoara, Romania; 6 Faculty of Bioengineering of Animal Resources, Discipline of Physiology, University of Life Sciences “King Mihai I”, 300645 Timișoara, Romania; marioaranicula@usvt.ro; 7 Department III of Functional Sciences—Biostatistics and Medical Informatics, “Victor Babeș” University of Medicine and Pharmacy, 300041 Timișoara, Romania; mirela.frandes@umft.ro

**Keywords:** diabetes distress, type 2 diabetes, mental health, older adults, lifespan perspective, Diabetes Distress Scale, educational level, multidisciplinary approaches, preventive strategies, psychosocial interventions

## Abstract

Background: Diabetes distress is a key mental health dimension of type 2 diabetes (T2D) and a growing concern as populations age, yet its determinants in older adults remain insufficiently characterized. The independent contributions of demographic and metabolic factors to severe distress and their implications for multidisciplinary screening across the lifespan remain unclear. Methods: In this cross-sectional study, we assessed 453 adults with T2D using the 17-item Diabetes Distress Scale (DDS-17). We categorized participants as experiencing low (<2), moderate (2–2.9), or severe (≥3) distress. We compared categories using the Kruskal–Wallis H test and chi-square tests. Logistic regression models were constructed with severe distress (DDS ≥ 3) as the outcome, adjusting for sex, educational level, and HbA1c. Results: Severe distress was identified in 19 participants (4.2%). Age differed significantly across DDS categories (Kruskal–Wallis H test, *p* = 0.003), with the severe group being significantly older (72.95 ± 4.38 years). Emotional burden was the dominant subscale across all groups. In multivariable logistic regression, increasing age (aOR = 1.163 per year; 95% CI: 1.055–1.282; *p* = 0.002) and lower educational level (high school vs. basic: aOR = 0.187; *p* = 0.020) were independently associated with severe distress, whereas metabolic variables were not independently associated with severe distress. ROC analysis identified age ≥ 70 years as an exploratory, sample-derived cut-off (AUC = 0.726, 95% CI 0.602–0.827; sensitivity 84%, specificity 55%). Results were robust in sensitivity analyses that excluded patients with diabetes duration < 5 years. Conclusions: Severe diabetes distress is more strongly associated with demographic vulnerability than with metabolic burden. Age ≥ 70 years and lower educational attainment may help identify older adults at increased risk of diabetes-related distress, supporting targeted psychological screening strategies in this lifespan stage.

## 1. Introduction

Diabetes mellitus (DM) is a chronic condition that affects millions globally [1,2]. Although medical care frequently concentrates on blood sugar control and physical issues, the emotional and mental challenges of managing diabetes are just as important. The Diabetes Distress Scale (DDS) is a validated tool that assesses emotional distress associated with diabetes management. It is increasingly recognized as an essential resource in clinical settings and research for identifying patients who may require psychological assistance [3].

Diabetes distress refers to the emotional stress and concerns associated with managing diabetes. Although distinct from clinical depression, it can similarly affect self-care, treatment adherence, and blood sugar levels [4,5]. Causes of diabetes distress include fear of complications, feeling that one has failed to control glucose, interpersonal challenges related to diabetes, and frustration with healthcare systems. Fisher and colleagues developed the DDS in 2005 to address the need for a reliable, valid measure of diabetes-specific emotional distress. In its full form (DDS-17), the instrument consists of 17 items grouped into four subscales: emotional burden, concerns about the demands of living with diabetes; physician-related distress, dissatisfaction with healthcare providers; regimen distress, frustration with the diabetes management regimen; and interpersonal distress, lack of support from friends and family [6].

Research shows that high levels of diabetes distress are linked to poorer glycemic control, reduced self-management, and lower quality of life [7,8]. However, this association is likely bidirectional and remains incompletely resolved. Elevated distress may erode self-care behaviors and thereby worsen glycemic control, whereas deteriorating control, hypoglycemic episodes, and new complications may, in turn, intensify emotional burden. Longitudinal cohorts and intervention trials provide evidence for effects operating in both directions, so that distress is best regarded as both a determinant and a consequence of impaired self-management. Cross-sectional designs such as the present one quantify these associations but cannot establish their direction, a constraint that applies to all findings reported here. Unlike depression scales, the DDS offers diabetes-specific insights, enabling healthcare providers to tailor interventions more precisely. Regular DDS screening can improve patient outcomes by identifying individuals who may benefit from targeted psychological or educational support. Incorporating the DDS into routine diabetes care can enhance holistic patient management. Clinicians can use it to initiate conversations about emotional well-being, identify stressors, and design individualized care plans that address both the medical and psychosocial aspects of diabetes.

The relative contributions of metabolic versus demographic factors to diabetes distress nevertheless remain unclear [9]. Moreover, age and educational level may influence distress through distinct mechanisms: older patients may face greater accumulated disease burden and reduced adaptive coping resources, while lower educational attainment may impair health literacy and self-management capacity. Beyond total distress scores, the DDS subscale profile, which comprises emotional burden, physician-related distress, regimen-related distress, and interpersonal distress, may reveal which dimensions drive clinical severity. Furthermore, Romanian or Eastern European populations lack a clinically actionable age threshold for screening. This study aimed to: (i) examine whether age (per year) and educational level are independently associated with severe diabetes distress in adults with T2D; (ii) characterize the DDS subscale profile across distress severity groups; (iii) determine the discriminative capacity of age using ROC analysis and identify an optimal clinical cut-off; and (iv) explore whether the associations of age and educational level with severe distress are modified by sex, using interaction terms and sex-stratified subgroup analyses.

## 2. Materials and Methods

### 2.1. Study Design and Participants

This cross-sectional study included 453 adults diagnosed with T2D. We defined inclusion and exclusion criteria according to clinical standards. To achieve the proposed objective, we conducted a descriptive, cross-sectional epidemiological survey using a questionnaire that assessed patients’ feelings toward the disease, how often they checked their blood sugar levels, their diabetes routine, family support, their relationship with their doctor, and their motivation to continue managing their diabetes. We administered the questionnaires to 453 patients aged 18 years or older admitted to the Diabetes, Nutrition and Metabolic Diseases Clinic of the Timișoara County Emergency Clinical Hospital between July and September 2025. All eligible patients invited during this period agreed to take part, and no patient declined, corresponding to a response rate of 100% (453/453). Because there were no non-responders, a formal non-responder analysis was not applicable, and selection bias arising from differential participation is therefore unlikely in this consecutively recruited clinic-based sample.

Study inclusion criteria: patients with T2D, diagnosed for at least 3 months. Exclusion criteria from the study: patients with type 1 diabetes, mental illness, cancer, gestational diabetes, pregnant women with T1D or T2D, untreated hypothyroidism, and those who did not want to participate in the study.

### 2.2. Clinical and Sociodemographic Variables

Variables included age (years), sex, diabetes duration (years), HbA1c (%), BMI (kg/m^2^), treatment type, and educational level. Glycated hemoglobin was quantified by immunoturbidimetry using an assay traceable to the National Glycohemoglobin Standardization Program (NGSP) and aligned with the Diabetes Control and Complications Trial (DCCT) reference method. Hoffmann-La Roche Ltd. (Basel, Switzerland) supplied the kit, which, according to the manufacturer’s specifications, has an inter-measurement coefficient of variation of 1.64%. The reference range was 4.8–6.4% [10].

Plasma glucose was determined enzymatically by a β-glucosidase-based method. Fasting concentrations between 70 and 110 mg/dL and postprandial concentrations below 140 mg/dL were regarded as normal, whereas diabetes was defined by a fasting value of at least 126 mg/dL or a postprandial value of at least 200 mg/dL [11].

We obtained blood pressure (BP) readings from the left upper arm with participants seated after 5–10 min of rest, using a conventional sphygmomanometer (Disytest, Jungingen, Germany) fitted with a cuff of appropriate bladder size. Systolic BP (SBP) was taken as the mean of two readings recorded within a 10 min interval. Participants were then categorized according to the 2018 ESC/ESH guidelines as having optimal (<120 systolic and <80 diastolic), normal (120–129 and/or 80–85), or high-normal (130–139 and/or 85–89) BP, or grade I (140–159 and/or 90–99), grade II (160–179 and/or 100–109), or grade III (≥180 and/or ≥110) hypertension [12].

### 2.3. Psychological Assessment

Diabetes-related distress was evaluated with the Diabetes Distress Scale (DDS). Severe distress was defined as DDS ≥ 3. The Diabetes Distress Scale-17 (DDS-17) questionnaire assessed distress among patients with T2D. The questionnaire included 17 questions that gathered various situations a person with diabetes may have experienced in the last few months that could have bothered them. They had 6 answer options available. If the situation was not distressing, they circled letter A (1 point); the more distressing the situation, the higher the letter they circled, up to F (6 points). To calculate the distress score in diabetic patients (DDS-17), which reflects the patient’s total stress, the points from the answers were added and divided by the number of questions [13], yielding a score. Totals below 2 were classified as little or no distress, values from 2 to 2.9 as moderate distress, and values of 3.0 or above as high distress [14]. We administered the full 17-item version of the instrument (DDS-17), yielding a total score and four subscale scores; we did not use the abbreviated two-item screener (DDS-2) in this study.

The Diabetes Distress Scale contains four domains: emotional burden (EB), physician-related distress (PRD), regimen-related distress (RRD), and interpersonal distress (IPD). To calculate emotional stress, add the scores for questions 1, 3, 8, 11, and 14, then divide by 5. To calculate stress related to the doctor, add questions 2, 4, 9, and 15, and divide the result by 4. To calculate regimen-related stress, add questions 5, 6, 10, 12, and 16, then divide the result by 5. To calculate interpersonal stress, add the scores for questions 7, 13, and 17, then divide the result by 3. For each domain, a score of <2 is considered little or no distress, a score of 2 to 2.9 moderate distress, and a score ≥ 3.0 high distress.

### 2.4. Statistical Analysis

We reported continuous data as mean ± standard deviation (SD) when the distribution approximated normality and as median with interquartile range (IQR) when skewed; we reported categorical variables as absolute frequencies and percentages (*n*, %). We evaluated distributional assumptions for parametric tests by visually inspecting histograms and Q–Q plots and using the Shapiro–Wilk test, and assessed homogeneity of variance with Levene’s test. Because preliminary distributional checks indicated departures from normality for the continuous variables of interest, differences across the three DDS severity categories (low, moderate, severe) were assessed primarily using the non-parametric Kruskal–Wallis H test, with Dunn’s post hoc correction for pairwise comparisons. One-way ANOVA followed by Tukey’s honestly significant difference (HSD) procedure was retained as a parametric sensitivity analysis and yielded consistent inferences across all primary comparisons. Categorical variables were compared using Pearson’s chi-square test, with Fisher’s exact test substituted for cells with expected counts <5 (a common situation given the small severe-distress subgroup). The four DDS subscales, emotional burden, physician-related distress, regimen-related distress, and interpersonal distress, were compared across distress categories using the same Kruskal–Wallis H test framework. Spearman’s rank correlation coefficients (ρ) were computed among clinical variables (such as, age, BMI, HbA1c, diabetes duration, systolic blood pressure), the DDS total score, and individual subscale scores; correlation strengths were interpreted using conventional thresholds (|*ρ*| < 0.30 weak, 0.30–0.50 moderate, >0.50 strong) and significance was reported at *p* < 0.05, *p* < 0.01, and *p* < 0.001. We interpreted correlation results primarily by effect size rather than statistical significance alone. We reported the three conventional significance tiers (*p* < 0.05, *p* < 0.01, *p* < 0.001) alongside the coefficients so readers can assess evidence strength alongside association magnitude across the full correlation matrix, rather than relying on a single dichotomous cut-off. Because these correlations were exploratory and hypothesis-generating rather than confirmatory, we did not apply a formal multiple-comparisons correction, and coefficients should be interpreted accordingly.

We constructed multivariable logistic regression models with severe diabetes distress (DDS ≥ 3) as the binary dependent variable. We modeled age as a continuous variable (per 1-year increase) after confirming approximate linearity in the logit model. We specified three nested models to assess independent contributions: Model 1 (crude) evaluated the unadjusted association of age with severe distress; Model 2 added educational level (categorical, with basic education as the reference) and sex; Model 3 additionally included HbA1c. Additional exploratory models incorporating BMI, diabetes duration, and treatment type did not materially alter the observed associations. We assessed multicollinearity among predictors using variance inflation factors, with all values below the conventional threshold of 5. Odds ratios (OR) and adjusted odds ratios (aOR) with 95% confidence intervals (CI) were reported for each predictor. We evaluated the discriminative ability of age as a continuous predictor of severe distress using receiver operating characteristic (ROC) analysis. We computed the area under the ROC curve (AUC) and estimated its 95% confidence interval using non-parametric bootstrap resampling (1000 iterations). As a sensitivity analysis addressing the small number of severe-distress events, we refitted the fully adjusted model using Firth’s penalized-likelihood logistic regression, which reduces small-sample bias in rare-event settings. The optimal age cut-off was determined using the Youden index (J = sensitivity + specificity − 1), which maximizes the balance between sensitivity and specificity and provides a clinically interpretable single threshold for screening. Robustness of the primary findings was tested through (i) a pre-specified sensitivity analysis excluding patients with diabetes duration < 5 years to ensure that effects were not driven by recently diagnosed individuals, and (ii) subgroup analyses stratified by sex and educational level. We included two-way interaction terms (age × sex; age × educational level) in the multivariable models to assess whether these variables modified the independent effect of age on severe distress. We addressed missing values using complete-case analysis, given the low proportion of missing data (<2% per variable) and the absence of evidence for non-random missingness. All statistical tests were two-sided, with statistical significance set at *p* < 0.05. Analyses were performed using R version 4.4.1 (R Foundation for Statistical Computing, Vienna, Austria) and MedCalc Statistical Software version 23.6.2 (MedCalc Software Ltd., Ostend, Belgium).

## 3. Results

### 3.1. Study Population Characteristics

We included 453 adults with T2D in the analysis. The cohort’s median age was 69 years (IQR 66–73), with a balanced sex distribution (241 females, 212 males). The median HbA1c was 8.5% (IQR 7.4–9.9), and the median BMI was 31.0 kg/m^2^ (IQR 27.0–35.0), indicating a predominantly overweight population. Most participants had long-standing diabetes (>10 years; *n* = 356), and most were retired (*n* = 406). Regarding educational level, participants were distributed across all categories, with the largest proportion having a high school education (*n* = 177). Severe diabetes distress (DDS ≥ 3) was identified in 19 participants (4.2%; 95% CI 2.7–6.5%), moderate distress in 114, and low distress in 320. Table 1 presents detailed baseline characteristics.

### 3.2. Differences Across DDS Categories

**Figure 1:**
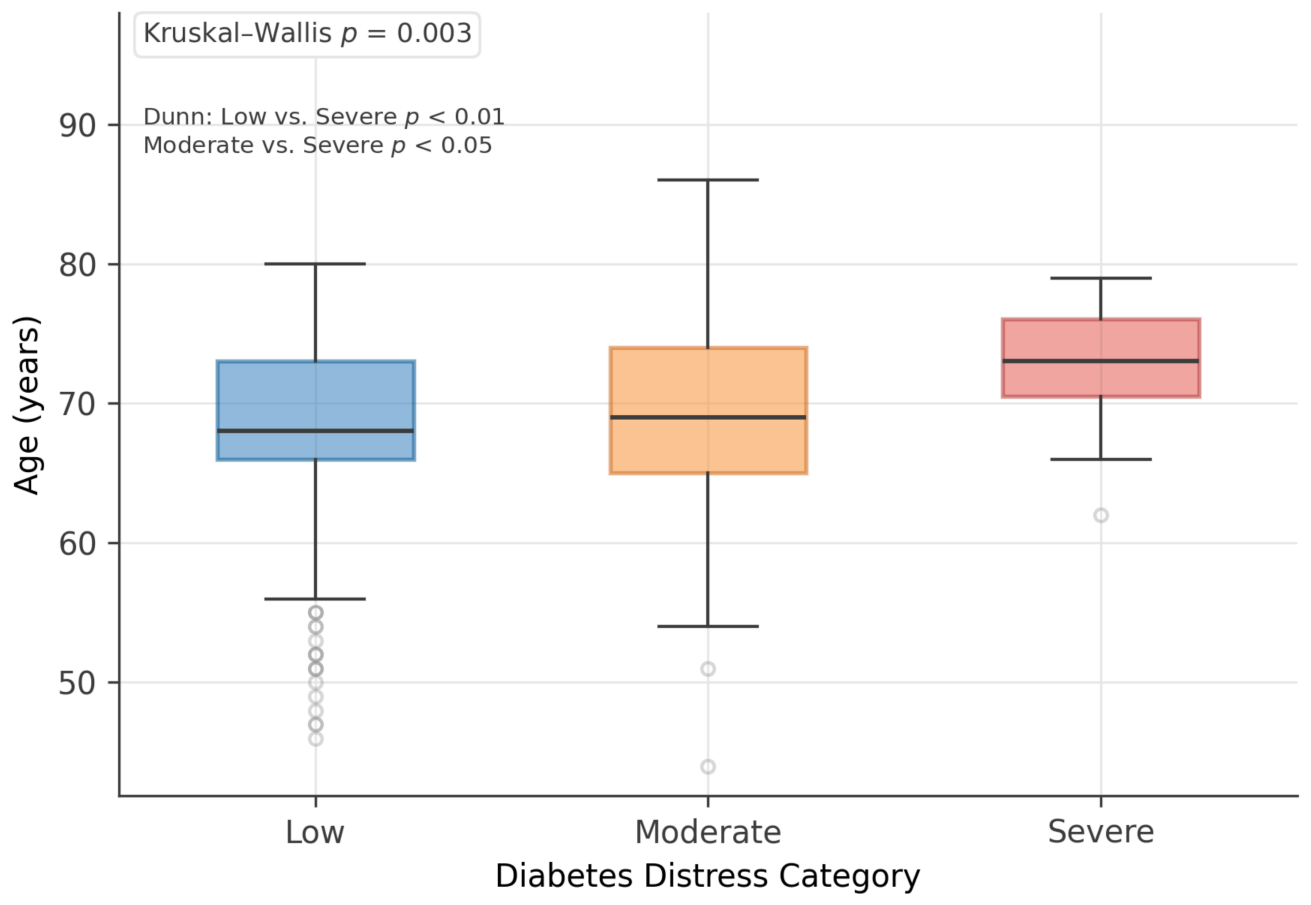
Distribution of age (years) across diabetes distress categories (Kruskal–Wallis, *p* = 0.003). Pairwise comparisons (Dunn): low vs. severe, *p* < 0.01; moderate vs. severe, *p* < 0.05. Boxes span the interquartile range, the horizontal line within each box indicates the median, whiskers extend to 1.5 × IQR, and open circles denote individual outliers; box colors identify the three distress categories (blue, low; orange, moderate; red, severe).

Age differed significantly across DDS categories (Kruskal–Wallis H test, *p* = 0.003; Figure 1). Pairwise comparisons (Dunn) showed that the severe-distress group was significantly older than both the low-distress (*p* < 0.01) and moderate-distress (*p* < 0.05) groups, whereas the low- and moderate-distress groups did not differ significantly. Sex distribution differed globally across DDS categories (χ^2^, *p* = 0.037). In contrast, HbA1c, BMI, and diabetes duration did not significantly differ across distress levels (all *p* > 0.05), indicating that metabolic parameters were not associated with distress severity at the descriptive level.

As shown in Table 2, age was the only continuous baseline variable that differed significantly across DDS categories, while body mass index, HbA1c, and diabetes duration were comparable between groups. Figure 1 visualizes the underlying age distribution and the magnitude of the upward shift in the severe-distress group.

**Figure 2:**
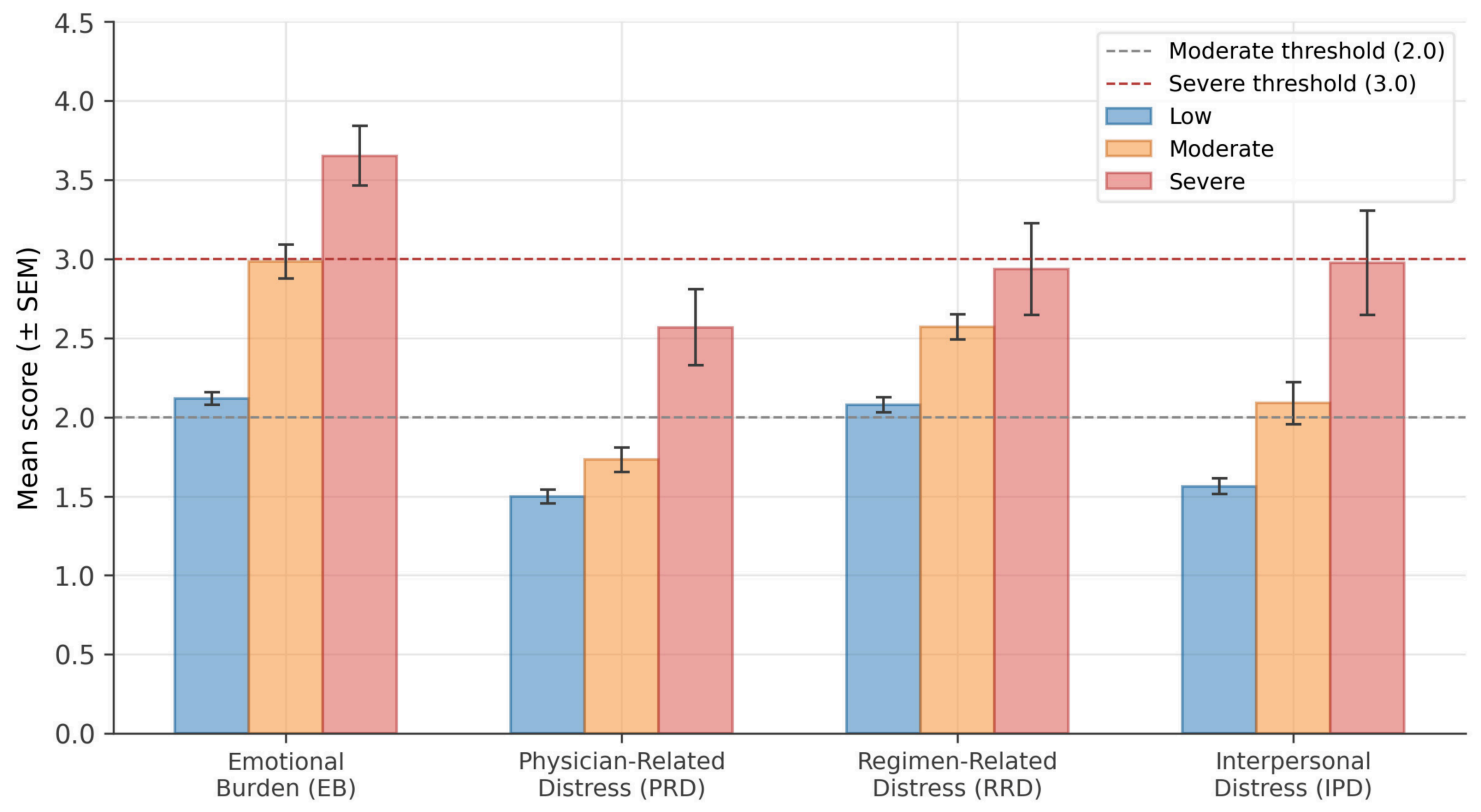
DDS subscales mean scores (±SEM) across distress categories. All four subscales differed significantly across groups (Kruskal–Wallis, all *p* < 0.001). Dashed lines indicate moderate (score 2.0) and severe (score 3.0) thresholds. EB = Emotional Burden; PRD = Physician-Related Distress; RRD = Regimen-Related Distress; IPD = Interpersonal Distress; SEM = standard error of the mean.

Analysis of DDS subscales revealed that all four domains differed significantly across distress categories (all Kruskal–Wallis H tests, *p* < 0.001; Figure 2). Emotional burden showed the highest mean scores across all groups (Low: 2.12 ± 0.71; Moderate: 2.98 ± 1.13; Severe: 3.65 ± 0.82) and was the only subscale that distinguished all three groups in pairwise comparisons (Dunn: Low vs. Moderate *p* < 0.001; Low vs. Severe *p* < 0.001; Moderate vs. Severe *p* < 0.05), suggesting that emotional burden may be the subscale most closely linked to increasing distress severity. Physician-related distress remained the lowest-scoring subscale across categories (Low: 1.50 ± 0.76; Moderate: 1.73 ± 0.82; Severe: 2.57 ± 1.04), suggesting that the patient–clinician relationship was generally not perceived as a major source of distress. Regimen-related distress did not differ significantly between the Moderate and Severe groups (Dunn test, *p* > 0.05), indicating that frustration with the treatment regimen contributes to overall burden but does not characterize the transition from moderate to severe distress.

**Figure 3:**
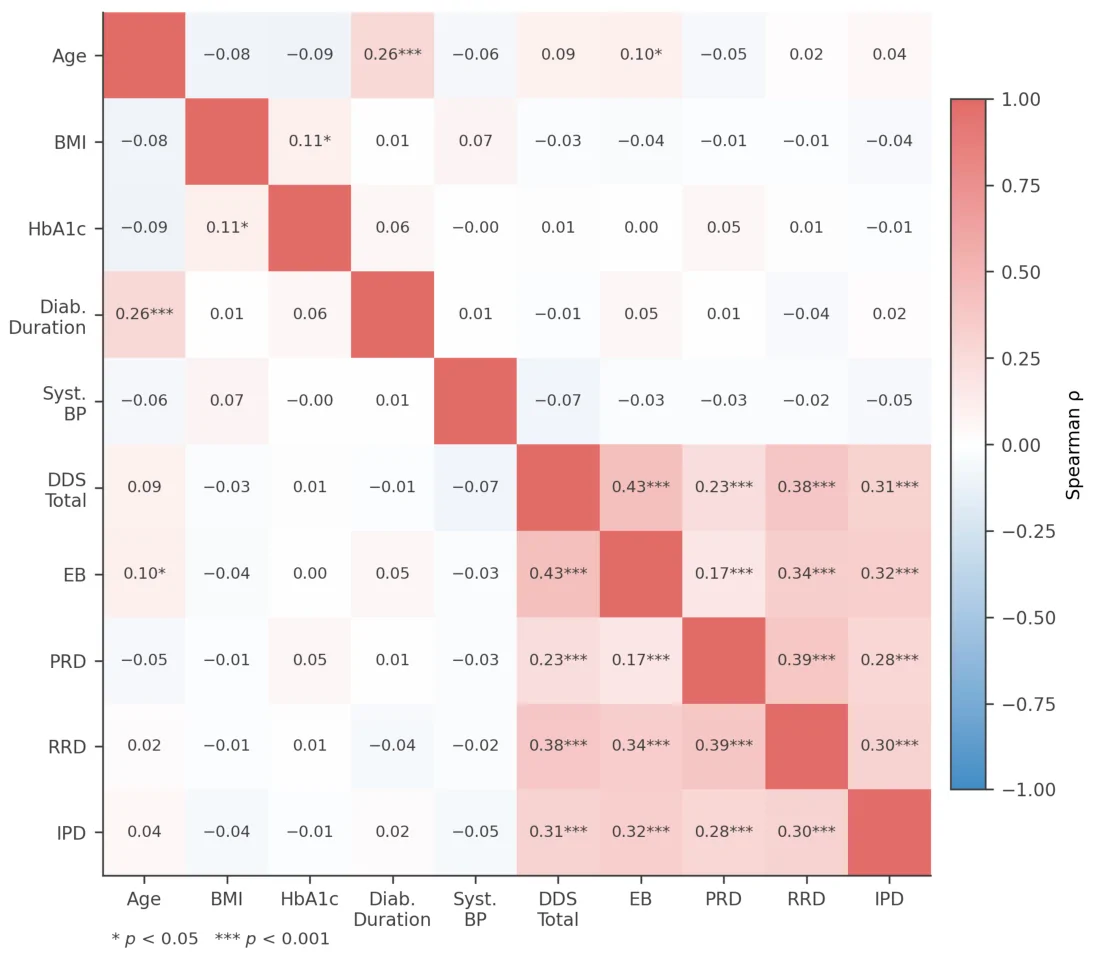
Spearman correlation matrix for key clinical and DDS variables. Asterisks denote statistical significance (* *p* < 0.05, *** *p* < 0.001). Metabolic parameters (HbA1c, BMI, diabetes duration) showed no significant correlation with the DDS total score, whereas all four DDS subscales were significantly intercorrelated.

To further characterize relationships among clinical, psychological, and demographic variables, we performed Spearman’s rank correlation analysis (Figure 3). All four DDS subscales were significantly correlated with the total DDS score (Emotional Burden ρ = 0.43, Regimen-related ρ = 0.38, Interpersonal ρ = 0.31, Physician-related ρ = 0.23; all *p* < 0.001) and were moderately intercorrelated. In contrast, none of the metabolic parameters (HbA1c, BMI, diabetes duration) or systolic blood pressure correlated significantly with total DDS score, reinforcing the dissociation between psychological and metabolic dimensions observed in the categorical analysis.

### 3.3. Discriminative Ability of Age: ROC Analysis and Clinical Cut-Off

**Figure 4:**
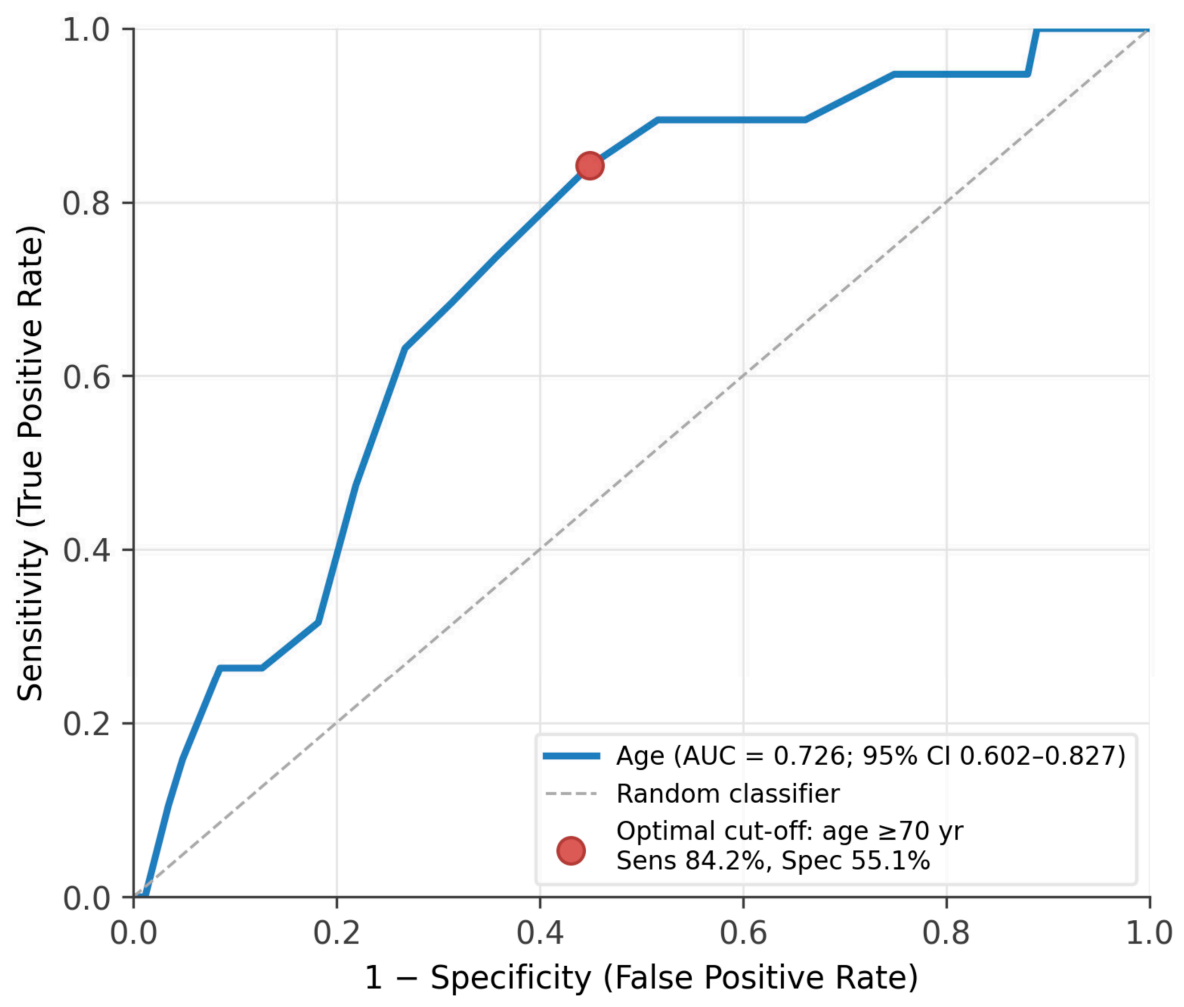
Receiver operating characteristic (ROC) curve for age as a predictor of severe diabetes distress (AUC = 0.726; 95% CI 0.602–0.827). The red dot marks the optimal Youden cut-off (age ≥ 70 years; sensitivity, 84.2%; specificity, 55.1%).

**Figure 5:**
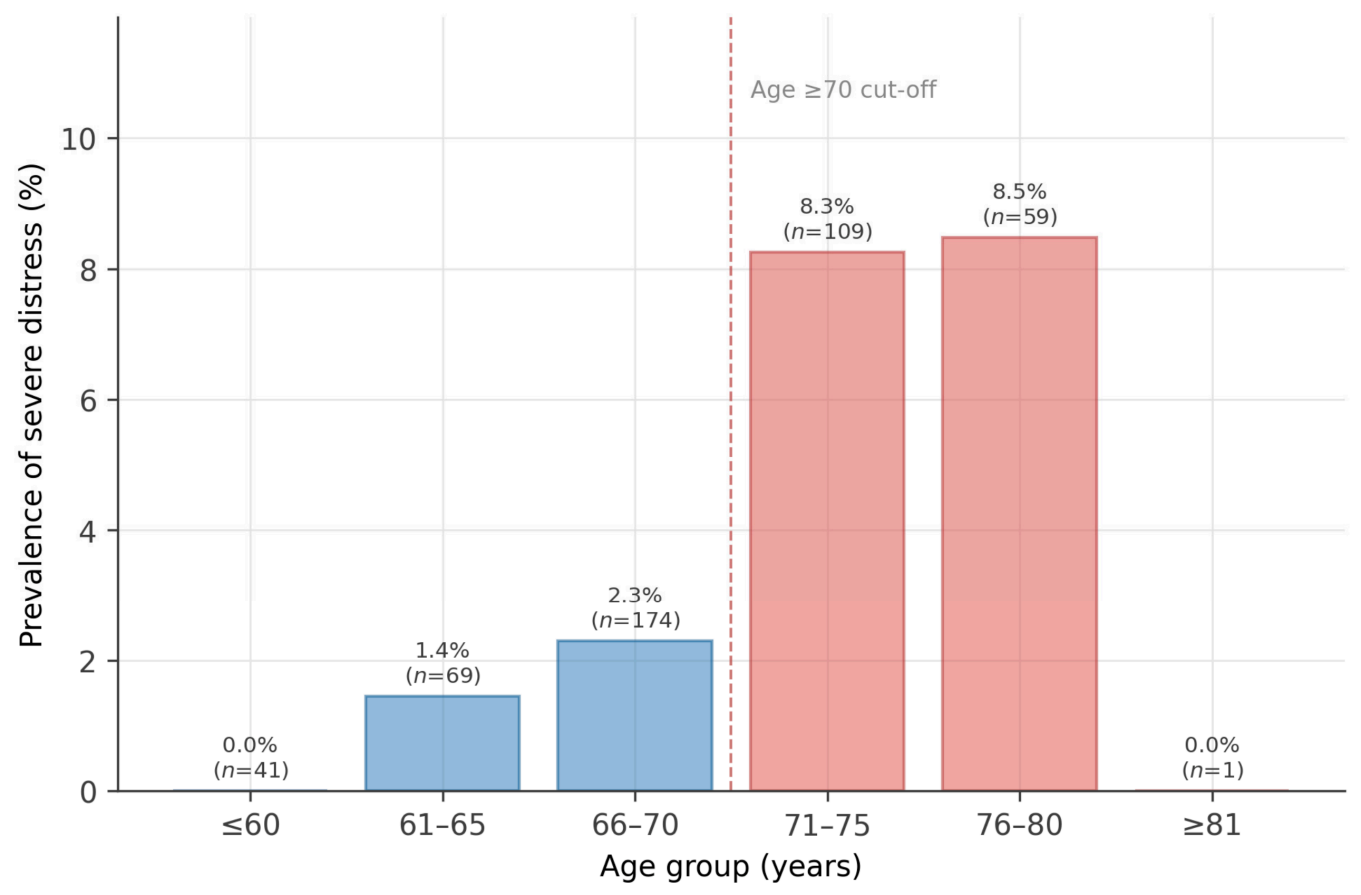
Prevalence of severe diabetes distress by age group. Bars shaded blue indicate age groups below, and bars shaded red age groups above, the identified cut-off of 70 years.

ROC analysis demonstrated that age had a moderate-to-good discriminative capacity for severe diabetes distress, with an area under the curve (AUC) of 0.726 (95% CI 0.602–0.827, bootstrap with 1000 iterations). Using the Youden index, the optimal age cut-off was ≥70 years, yielding a sensitivity of 84.2% and a specificity of 55.1% (Figure 4). The distribution of severe distress prevalence by age group confirmed a marked increase above this threshold: prevalence rose from 1.4% in the 61–65-year-old group and 2.3% in the 66–70-year-old group to 8.3% in the 71–75-year-old group and 8.5% in the 76–80-year-old group, compared with 0% in participants aged ≤60 years (Figure 5). This non-linear pattern suggests that the risk of severe distress accelerates substantially after the age of 70 years [15]. This cut-off should, however, be regarded as exploratory rather than definitive. We derived it from the present sample by maximizing the Youden index, a procedure that selects the threshold best fitting these data and therefore tends to yield optimistic performance estimates; it rests on only 19 events; and it has not been tested in an independent cohort. External validation is required before the threshold could be considered for clinical use.

### 3.4. Subgroup Analysis: Sex and Educational Level

**Figure 6:**
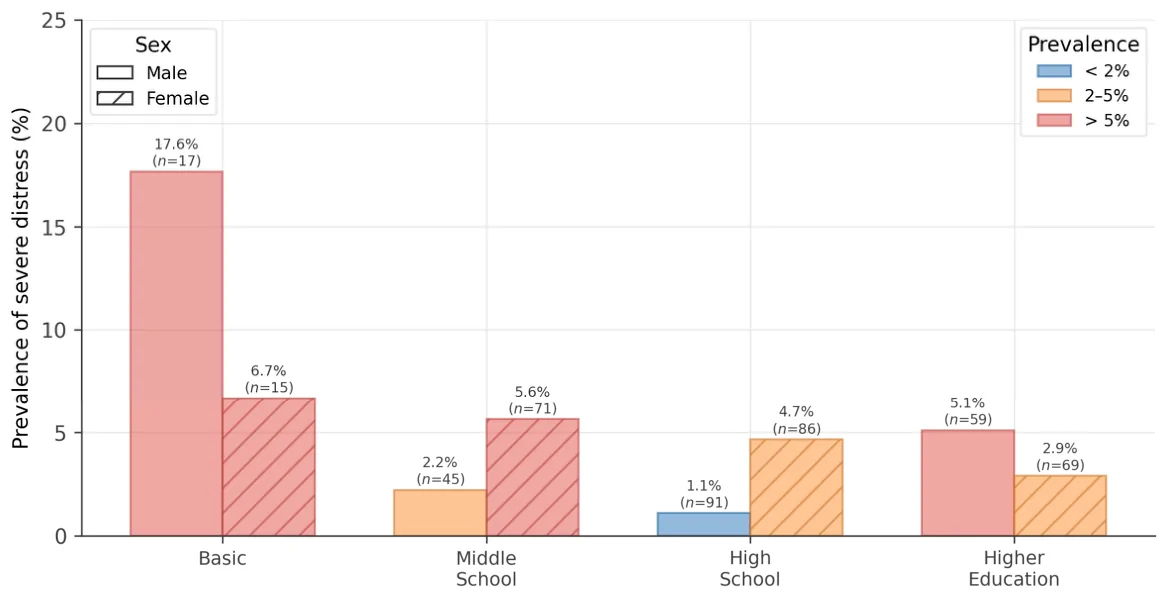
Prevalence of severe diabetes distress (DDS ≥ 3) by educational level, stratified by sex. Bars are color-coded by prevalence level (blue: <2%, orange: 2–5%, red: >5%). Numbers above bars indicate *n* per subgroup.

Subgroup analysis stratified by sex revealed divergent patterns in the relationship between educational level and severe distress (Figure 6). Among males, the basic education subgroup had the highest prevalence of severe distress (17.6%), with a marked gradient toward lower prevalence at higher education levels (middle school: 2.2%, high school: 1.1%, higher education: 5.1%). Among females, the distribution was more uniform across educational categories (basic: 6.7%, middle school: 5.6%, high school: 4.7%, higher education: 2.9%), without a clear gradient. These patterns suggest a possible sex-specific pattern that warrants confirmation in larger cohorts. Neither interaction term reached statistical significance. For age × sex, the interaction odds ratio was 1.095 (95% CI 0.897–1.337; *p* = 0.372; likelihood-ratio χ^2^(1) = 0.81, *p* = 0.367). For age × educational level, the three-degree-of-freedom likelihood-ratio test was likewise non-significant (χ^2^(3) = 3.07, *p* = 0.382), with individual interaction odds ratios, each relative to basic education, of 1.259 (95% CI 0.960–1.650; *p* = 0.096) for middle school, 1.176 (95% CI 0.902–1.532; *p* = 0.232) for high school, and 1.088 (95% CI 0.843–1.405; *p* = 0.518) for higher education. These estimates indicate that the independent effects of age and educational level on severe distress were statistically homogeneous across sex and educational strata, although the small number of events limits the power to detect effect modification.

### 3.5. Predictors of Severe Diabetes Distress: Multivariable Logistic Regression

We performed logistic regression analyses using severe distress (DDS ≥ 3) as the dependent variable. In unadjusted analysis, increasing age (per year) was associated with higher odds of severe distress (OR = 1.159 per year; 95% CI: 1.054–1.275; *p* = 0.002). After adjustment for educational level and sex, age remained independently associated with severe distress (aOR = 1.163 per year; 95% CI: 1.055–1.282; *p* = 0.002). Lower educational level was also independently associated with severe distress (high school vs. basic education: aOR = 0.187; 95% CI: 0.046–0.769; *p* = 0.020), whereas sex was not significantly associated (*p* = 0.911). Further adjustment for HbA1c did not materially change these associations. Age and educational level remained significant predictors, while HbA1c was not significantly associated with severe distress (Table 3). Additional exploratory models incorporating BMI, diabetes duration, and treatment type did not materially alter the observed associations.

**Figure 7:**
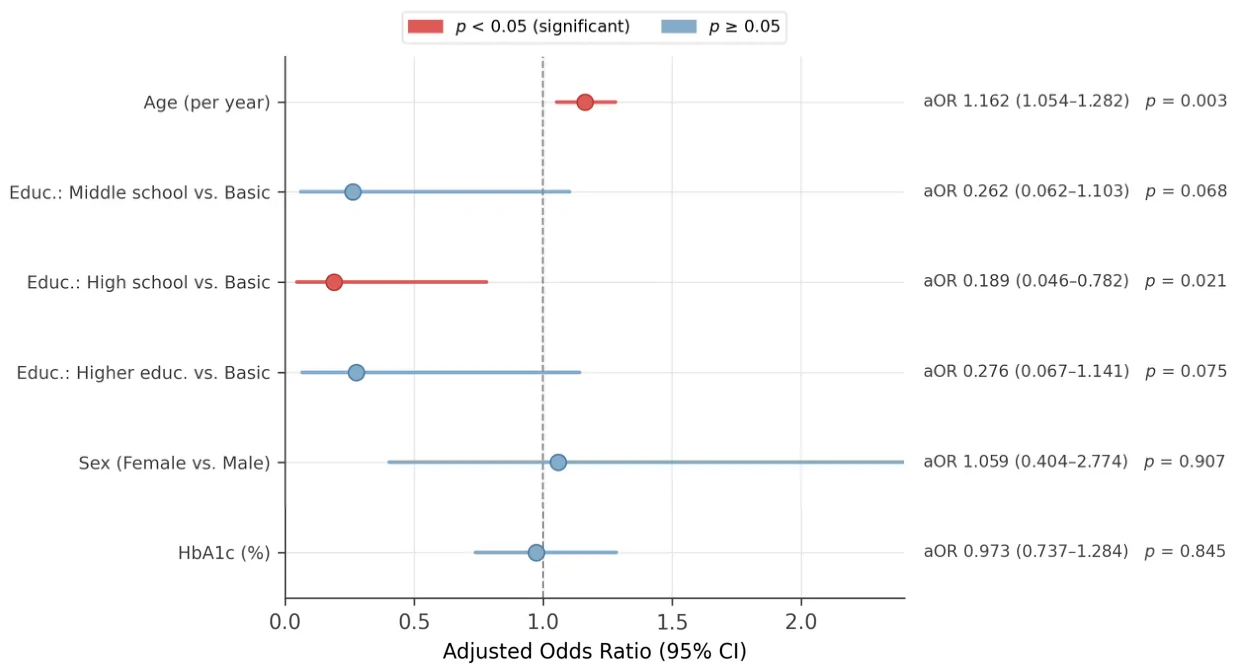
Forest plot of adjusted odds ratios (95% CI) from the fully adjusted logistic regression model (Model 3). Red markers indicate statistically significant associations (*p* < 0.05). The vertical dashed line marks an odds ratio of 1, indicating no association.

Figure 7 summarizes the adjusted odds ratios from the fully adjusted model (Model 3), where only age and educational level emerged as independently associated with severe distress, while HbA1c and sex-related estimates clustered around unity.

### 3.6. Robustness and Sensitivity Analyses

This cohort showed no evidence of nonlinearity in the association between age and severe distress across the age range. A sensitivity analysis excluding 29 patients with diabetes duration <5 years (*n* = 424) yielded virtually identical results: the association between age and severe distress remained significant and of similar magnitude (aOR = 1.155 per year; 95% CI: 1.049–1.272; *p* = 0.003), confirming that the findings are not driven by recently diagnosed patients. Interaction terms were not statistically significant (age × sex, *p* = 0.372; age × educational level, likelihood-ratio χ^2^(3) = 3.07, *p* = 0.382; Table 4), indicating that the independent effects were stable. Because age was modeled per 1-year increment, its confidence interval is necessarily narrow on that scale; the same estimate per decade corresponds to an aOR of 4.49 (95% CI 1.69–11.99), reflecting substantial uncertainty attributable to only 19 severe-distress events. To confirm that the estimates were not distorted by this small event count, Model 3 was refitted using Firth’s penalized-likelihood logistic regression; the results were materially unchanged (age aOR 1.154, 95% CI 1.054–1.263; high school vs. basic education aOR 0.194, 95% CI 0.051–0.739), indicating that the reported associations are not artifacts of sparse-data bias.

## 4. Discussion

This cross-sectional study of 453 adults with T2D found that severe diabetes distress (DDS ≥ 3) was independently associated with older age and lower educational attainment but not with metabolic control markers such as HbA1c, BMI, or diabetes duration. Three additional findings support this conclusion: (i) emotional burden was the primary DDS subscale and the only one distinguishing all three distress severity groups; (ii) ROC analysis identified age ≥70 years as an exploratory, sample-derived screening threshold (AUC = 0.726) that requires validation in independent cohorts; and (iii) a possible sex-specific pattern in the education–distress relationship that warrants confirmation in larger cohorts.

Our study identified clinically meaningful diabetes-related distress (DDS ≥ 2; moderate or severe) in 29.4% of participants and severe distress (DDS ≥ 3) in 4.2%. International comparisons require careful attention to the cut-off used. Applying the Fisher 2008 single-threshold criterion (DDS ≥ 3 = high distress) [6], our 4.2% rate is substantially lower than the 29.4% reported by Batais et al. in Saudi adults with T2D [16,17] and the 51.3% in a U.S. cohort using a comparable threshold [18]. When the broader Fisher 2012 cut-off (DDS ≥ 2 = clinically meaningful distress) [14] is applied, our 29.4% prevalence remains lower than reports from Malaysia (49.2%) [19], Bangladesh (52.5%) [20], China (43%) [21], and Canada (39%) [22], and below pooled South Asian estimates of 44% [23]. Studies using the Problem Areas in Diabetes (PAID) questionnaire have generally yielded lower prevalences (8.9–10.7% in German samples) [13], reflecting both methodological and population differences between PAID and DDS instruments [24]. Several factors likely contribute to the relatively low burden observed in our cohort: a sample predominantly composed of retired individuals who may report fewer occupational stressors, possible under-reporting of emotional distress, and culturally specific patterns in the expression of distress [25]. Two further, and probably more consequential, explanations deserve emphasis. First, patients with a known psychiatric diagnosis were excluded by design; because psychological comorbidity and diabetes distress frequently co-occur, this criterion is likely to have removed the subgroup at highest risk of severe distress and to have lowered the observed prevalence relative to studies that applied no such exclusion. Second, the cohort was recruited from a tertiary clinic where patients have long-standing disease (median 18 years) and continuous specialist follow-up; sustained professional contact and progressive adaptation to the condition may attenuate reported distress, consistent with the very low physician-related distress scores observed here. Differences in the linguistic and cultural adaptation of the DDS may further limit the comparability of absolute prevalence estimates across countries, as the Romanian version has not undergone formal psychometric validation. Taken together, these considerations suggest that our 4.2% figure should be read as a conservative, clinic-specific estimate rather than as evidence of a genuinely lower burden of distress in the Romanian population. Two quantitative considerations reinforce this reading. First, the severe-distress estimate rests on only 19 cases, so its binomial confidence interval is wide (2.7–6.5%) and overlaps the lower bound of several published series; part of the apparent gap therefore reflects sampling imprecision rather than a systematically lower burden. Second, the contrast narrows markedly when a common criterion is applied: using the DDS ≥ 2 threshold, clinically meaningful distress affected 29.4% of our cohort (95% CI 25.4–33.7%), which lies much closer to, although still below, the Malaysian, Bangladeshi, Chinese, and Canadian estimates. Taken together, this suggests that the divergence is driven largely by the choice of cut-off and cohort composition rather than by a genuinely distinct distress profile in Romanian patients.

Among the four DDS subscales, physician-related distress had the lowest mean score across all groups (1.50 in low, 1.73 in moderate, 2.57 in severe), whereas emotional burden had the highest (2.12, 2.98, 3.65, respectively), suggesting that the patient–clinician relationship was generally not perceived as a source of substantial distress in our cohort. This is consistent with reports describing emotional burden as the predominant DDS dimension across diverse populations [26,27]. Demographic variables, particularly educational level, helped identify the subgroup most affected by diabetes-related distress. A lower educational level was independently associated with greater distress in our study, consistent with reports from other settings in which limited health literacy may amplify the burden of diabetes [28,29] and with broader evidence that socioeconomic disadvantage shapes psychosocial outcomes in diabetes care [30].

Educational attainment in this context should not be read as an effect of formal schooling itself, but as a readily measured proxy for a cluster of related resources: the functional health literacy and numeracy required for self-monitoring and treatment adjustment, socioeconomic position and the material resources it affords, familiarity with the healthcare system, and the range of coping strategies available when disease demands intensify. Patients with limited education may therefore experience an identical clinical regimen as more burdensome and less controllable, which plausibly translates into greater emotional distress. Consistent with this interpretation, the association in our data was strongest for the contrast between basic and high-school education, whereas estimates for the intermediate and highest categories were imprecise and not significant; the gradient should therefore be regarded as directional rather than strictly monotonic. Practically, educational level is routinely recorded and requires no additional instrument, making it an efficient stratifying variable for identifying patients who may benefit from simplified educational materials and more intensive psychological support. The magnitude of the effect in our data underlines this point: relative to patients with only basic education, those who had completed high school had approximately one-fifth the odds of severe distress (aOR 0.192, 95% CI 0.045–0.809), and the remaining educational categories showed effects in the same direction although their intervals included unity. The subgroup analysis further indicates that the burden is concentrated in a specific profile: among men with basic education, the prevalence of severe distress reached 17.6%, more than four times the cohort average, whereas among women, the educational gradient was shallow. In interpreting our findings, this matters in two respects. It shows that the demographic signal we report is not a diffuse age effect but is concentrated where advanced age and limited education coincide, and it delineates a clinically recognizable group—older patients with limited schooling, particularly men—in whom the yield of distress screening is likely to be highest. Because educational attainment is fixed long before diagnosis, it cannot itself be a treatment target; its clinical value lies in stratifying who is screened and in adapting how information and support are delivered, rather than in whether they are offered at all.

In contrast to some prior reports, diabetes duration in our sample was not significantly associated with overall DDS score or severe distress in multivariable models (*p* = 0.321). This may reflect the relatively homogeneous distribution of long-standing disease in our cohort (median 18.0 years, IQR 12.0–24.0). Nevertheless, the subscale analysis showed that regimen-related and interpersonal distress increased progressively across severity groups, consistent with cumulative self-management burden. Notably, regimen-related distress did not differentiate moderate from severe cases (Dunn *p* > 0.05), suggesting it contributes to overall burden rather than to clinical severity escalation, a novel observation warranting replication. This is particularly interesting given prior longitudinal evidence that reductions in regimen-related distress are associated with improved diabetes self-management and glycemic control over time [31]. The sensitivity analysis excluding patients with disease duration <5 years confirmed the stability of all primary findings (aOR 1.155; *p* = 0.003).

Beyond demographic determinants, our multivariable analysis additionally adjusts for HbA1c, confirming that severe distress is dissociated from glycemic burden in this cohort (HbA1c *p* = 0.915). Additional exploratory models incorporating BMI, diabetes duration, and treatment type did not materially alter the observed associations. These null findings are most plausibly explained by the homogeneity of clinical profiles in our specialty-clinic sample, uniformly long disease duration (median 18.0 years, IQR 12.0–24.0), narrow HbA1c variation (median 8.5%, IQR 7.4–9.9), and a predominant overweight/obesity status, which restricts variance and reduces statistical power to detect metabolic–distress associations.

The dominance of emotional burden across all distress categories aligns with the literature characterizing diabetes distress as fundamentally an emotional experience rather than a problem of medical non-compliance. Emotional burden was the only subscale that differentiated among the three groups in the post hoc analysis, including the moderate vs. severe comparison, suggesting that it may be the subscale most closely linked to increased distress severity. This has direct clinical implications: interventions targeting emotional burden, such as problem-solving therapy, acceptance-based approaches, or structured peer-support programs, have been proposed as candidate strategies for preventing progression to severe distress [26,32]; the present cross-sectional design cannot evaluate their effectiveness, and this therefore remains a hypothesis for prospective testing. The AUC of 0.726 for age as a predictor of severe distress is comparable to other demographic screening tools in diabetes management. The identified cut-off of 70 years, with a sensitivity of 84.2%, would capture most severe cases, although its modest specificity of 55.1% means that a substantial proportion of patients without severe distress would also screen positive [33]. In clinical practice, and pending confirmation in independent cohorts, this threshold might serve as a low-burden prompt for considering DDS screening, particularly in primary care or outpatient diabetes clinics serving older adults. Prior Romanian evidence has similarly documented a considerable psychological symptom burden among patients with diabetes evaluated in a specialist clinic setting, reinforcing the case for routine mental-health assessment as part of diabetes care [34]. The subgroup analysis revealed that the educational gradient was more pronounced in males (basic education: 17.6% vs. higher education: 5.1%) than in females (basic education: 6.7% vs. higher education: 2.9%), suggesting a possible sex-specific pattern that warrants confirmation in larger cohorts. 

Data collection was conducted at a single public hospital, which may not fully represent other centers in Romania. Participants were recruited from specific clinics or regions, limiting generalizability to the broader Romanian population with T2D. The cross-sectional design can establish only associations, not causal relationships, between distress and associated factors. Some variables (e.g., psychological symptoms, lifestyle behaviors) were measured via self-report, which may be subject to recall bias or social desirability. Factors such as socioeconomic status, diabetes self-management education, or comorbid mental health disorders may not have been fully accounted for. Laboratory and clinical indicators (e.g., HbA1c, BMI) were measured once, which may not reflect long-term status. The study identifies distress but does not explore in depth the subjective experiences or cultural factors underlying it. In addition, the small number of severe-distress events (*n* = 19) relative to the number of covariates yielded a low events-per-variable ratio in the multivariable models, which may reduce the stability of the coefficient estimates and widen their confidence intervals; these associations should therefore be interpreted with caution and confirmed in larger samples. Several limitations specific to the measurement instrument also merit comment. The DDS-17 is a self-report screening questionnaire rather than a diagnostic tool: it quantifies perceived distress at a single point in time, cannot distinguish transient reactions from persistent states, and does not differentiate diabetes distress from overlapping constructs such as depression or anxiety, neither of which was assessed here. Its fixed four-domain structure may not capture culturally salient sources of distress such as financial strain or caregiving obligations, and the Romanian-language version has not been formally validated psychometrically in this population. Future work should therefore prioritize the formal validation of the Romanian DDS, longitudinal designs capable of clarifying the temporal ordering of distress and glycemic deterioration, and concurrent administration of depression and anxiety measures to delineate construct boundaries. Multicenter recruitment including primary care and non-specialist settings would improve generalizability, while digital and ecological momentary assessment could capture fluctuations that single administrations inevitably miss. Ultimately, intervention trials targeting emotional burden in older adults with limited educational attainment, the subgroup identified here as most vulnerable, are needed to establish whether screening translates into improved clinical and psychological outcomes.

## 5. Conclusions

Severe diabetes distress in adults with T2D is more strongly associated with demographic vulnerability, particularly older age and lower educational attainment, than with metabolic burden. Emotional concerns, rather than treatment-regimen frustration or healthcare-provider relationships, appear to drive the transition from moderate to severe distress. These findings are consistent with a case for targeted psychological screening in older adults with T2D and limited educational attainment, with particular attention to those at the intersection of advanced age and reduced health literacy. The present data cannot determine whether interventions targeting emotional burden, such as problem-solving therapy, acceptance-based approaches, or structured peer-support programs, reduce progression to severe distress; prospective, ideally randomized, studies within a multidisciplinary care model should examine this. The dissociation between metabolic control indicators and distress severity reinforces the view that diabetes distress is a distinct clinical construct that warrants independent screening alongside conventional glycemic monitoring across the lifespan. Because this design is cross-sectional, all reported associations are non-causal, and the proposed age threshold is exploratory and awaits external validation.

## Figures and Tables

**Table 1:** Baseline characteristics of the study population (*n* = 453).

Variable	Total (*n* = 453)
Age (years)	69.0 (66.0–73.0)
Female, n (%)	241 (53.2%)
Male, n (%)	212 (46.8%)
Diabetes duration (years)	18.0 (12.0–24.0)
HbA1c (%)	8.5 (7.4–9.9)
BMI (kg/m^2^)	31.0 (27.0–35.0)
Educational level, n (%)	
Basic	32 (7.1%)
Middle school	116 (25.6%)
High school	177 (39.1%)
Higher education	128 (28.3%)
Diabetes distress, n (%)	
Low (<2)	320 (70.6%)
Moderate (2–2.9)	114 (25.2%)
Severe (≥3)	19 (4.2%)

Data are presented as median (IQR) for continuous variables and n (%) for categorical variables. Abbreviations: BMI = body mass index; HbA1c = glycated hemoglobin; IQR = interquartile range.

**Table 2:** Comparison of clinical and demographic variables across DDS categories.

Variable	Low (*n* = 320)	Moderate (*n* = 114)	Severe (*n* = 19)	*p*-Value
Age (years)	68.0 (66.0–73.0)	69.0 (65.0–74.0)	73.0 (70.5–76.0)	0.003
Sex (female), n (%)	158 (49.4%)	72 (63.2%)	11 (57.9%)	0.037
BMI (kg/m^2^)	30.8 (26.7–35.4)	31.0 (27.4–35.0)	29.1 (26.5–32.6)	0.295
Diabetes duration (years)	18.0 (12.0–24.0)	18.0 (12.2–25.0)	18.0 (14.5–22.5)	0.626
HbA1c (%)	8.6 (7.2–10.0)	8.5 (7.6–9.8)	8.2 (7.4–9.3)	0.714
Education level, n (%)				0.024
Basic	19 (5.9%)	9 (7.9%)	4 (21.1%)	
Middle school	81 (25.3%)	30 (26.3%)	5 (26.3%)	
High school	118 (36.9%)	54 (47.4%)	5 (26.3%)	
Higher education	102 (31.9%)	21 (18.4%)	5 (26.3%)	

Data are presented as median (IQR) for continuous variables and n (%) for categorical variables. *p*-values were obtained from the Kruskal–Wallis H test (continuous variables) and Pearson’s chi-square test (categorical variables). Abbreviations: BMI = body mass index; DDS = Diabetes Distress Scale; HbA1c = glycated hemoglobin; IQR = interquartile range.

**Table 3:** Logistic regression models for severe diabetes distress (DDS ≥ 3) as the dependent variable. Model 1: crude association with age. Model 2: adjusted for age, educational level, and sex. Model 3: additionally adjusted for HbA1c.

Variable	Model 1 OR (95% CI)	Model 2 aOR (95% CI)	Model 3 aOR (95% CI)	*p*-Value (M3)
Age (per year)	1.159 (1.054–1.275)	1.163 (1.055–1.282)	1.180 (1.060–1.313)	0.002
Education (ref: basic)				
Middle school	—	0.261 (0.062–1.096)	0.273 (0.065–1.153)	0.077
High school	—	0.187 (0.046–0.769)	0.192 (0.045–0.809)	0.025
Higher education	—	0.274 (0.066–1.130)	0.276 (0.066–1.153)	0.078
Sex (female)	—	1.056 (0.403–2.766)	0.999 (0.378–2.645)	0.999
HbA1c (%)	—	—	0.985 (0.747–1.300)	0.915

OR = odds ratio; aOR = adjusted odds ratio; CI = confidence interval; — = not included in model.

**Table 4:** Interaction terms added to the fully adjusted logistic regression model (Model 3) for severe diabetes distress.

Interaction Term	OR	95% CI	*p*-Value
Age × sex (female vs. male)	1.095	0.897–1.337	0.372
Age × middle school (vs. basic)	1.259	0.960–1.650	0.096
Age × high school (vs. basic)	1.176	0.902–1.532	0.232
Age × higher education (vs. basic)	1.088	0.843–1.405	0.518

Each interaction term was added separately to Model 3. Likelihood-ratio tests: age × sex χ^2^(1) = 0.81, *p* = 0.367; age × educational level χ^2^(3) = 3.07, *p* = 0.382. We centered age at the cohort mean. OR = odds ratio; CI = confidence interval.

## Data Availability

The data presented in this study are available on request from the corresponding author. Access is restricted because the dataset contains sensitive patient health information and is subject to the ethical approval conditions granted by the Institutional Ethics Committee, which limits open dissemination to protect participant confidentiality. Please direct data access requests to the corresponding authors.

## Abbreviations

The following abbreviations are used in this manuscript:

aOR	Adjusted odds ratio
AUC	Area under the receiver operating characteristic curve
BMI	Body mass index
BP	Blood pressure
CI	Confidence interval
DDS	Diabetes Distress Scale
DM	Diabetes mellitus
EB	Emotional burden (DDS subscale)
HbA1c	Glycated hemoglobin A1c
IDF	International Diabetes Federation
IPD	Interpersonal distress (DDS subscale)
IQR	Interquartile range
NGSP	National Glycohemoglobin Standardization Program
OR	Odds ratio
PAID	Problem Areas in Diabetes
PRD	Physician-related distress (DDS subscale)
ROC	Receiver operating characteristic
RRD	Regimen-related distress (DDS subscale)
SEM	Standard error of the mean
T2D	Type 2 diabetes

## References

[1] Sun H., Saeedi P., Karuranga S., Pinkepank M., Ogurtsova K., Duncan B.B., Stein C., Basit A., Chan J.C.N., Mbanya J.C. (2022). IDF Diabetes Atlas: Global, regional and country-level diabetes prevalence estimates for 2021 and projections for 2045. Diabetes Res. Clin. Pract..

[2] GBD 2021 Diabetes Collaborators (2023). Global, regional, and national burden of diabetes from 1990 to 2021, with projections of prevalence to 2050: A systematic analysis for the Global Burden of Disease Study 2021. Lancet.

[3] Polonsky W.H., Fisher L., Earles J., Dudl R.J., Lees J., Mullan J., Jackson R.A. (2005). Assessing psychosocial distress in diabetes: Development of the Diabetes Distress Scale. Diabetes Care.

[4] Fisher L., Skaff M.M., Mullan J.T., Arean P., Glasgow R., Masharani U. (2007). Clinical depression versus distress among patients with type 2 diabetes: Not just a question of semantics. Diabetes Care.

[5] Gonzalez J.S., Fisher L., Polonsky W.H. (2011). Depression in diabetes: Have we been missing something important?. Diabetes Care.

[6] Fisher L., Glasgow R.E., Mullan J.T., Skaff M.M., Polonsky W.H. (2008). Development of a brief diabetes distress screening instrument. Ann. Fam. Med..

[7] Fisher L., Mullan J.T., Arean P., Glasgow R.E., Hessler D., Masharani U. (2010). Diabetes distress but not clinical depression or depressive symptoms is associated with glycemic control in both cross-sectional and longitudinal analyses. Diabetes Care.

[8] Fisher L., Glasgow R.E., Strycker L.A. (2010). The relationship between diabetes distress and clinical depression with glycemic control among patients with type 2 diabetes. Diabetes Care.

[9] Perrin N.E., Davies M.J., Robertson N., Snoek F.J., Khunti K. (2017). The prevalence of diabetes-specific emotional distress in people with type 2 diabetes: A systematic review and meta-analysis. Diabet. Med..

[10] Little R.R., Rohlfing C.L., Sacks D.B., NGSP Scientific Advisory Committee (2011). Status of hemoglobin A1c measurement and goals for improvement: From chaos to order for improving diabetes care. Clin. Chem..

[11] American Diabetes Association Professional Practice Committee (2024). 2. Diagnosis and Classification of Diabetes: Standards of Care in Diabetes—2024. Diabetes Care.

[12] Williams B., Mancia G., Spiering W., Agabiti Rosei E., Azizi M., Burnier M., Clement D.L., Coca A., de Simone G., Dominiczak A. (2018). 2018 ESC/ESH Guidelines for the management of arterial hypertension. Eur. Heart J..

[13] Hermanns N., Kulzer B., Krichbaum M., Kubiak T., Haak T. (2005). Affective and anxiety disorders in a German sample of diabetic patients: Prevalence, comorbidity and risk factors. Diabet. Med..

[14] Fisher L., Hessler D.M., Polonsky W.H., Mullan J. (2012). When is diabetes distress clinically meaningful? Establishing cut points for the Diabetes Distress Scale. Diabetes Care.

[15] Helgeson V.S., Van Vleet M., Zajdel M. (2020). Diabetes stress and health: Is aging a strength or a vulnerability?. J. Behav. Med..

[16] Batais M.A., Alfraiji A.F., Alyahya A.A., Aloofi O.A., Almashouq M.K., Alshehri K.S., Almizel A.M., Alotaibi M.T., Alosaimi F.D. (2021). Assessing the prevalence of diabetes distress and determining its psychosocial predictors among Saudi adults with type 2 diabetes: A cross-sectional study. Front. Psychol..

[17] Aljuaid M.O., Almutairi A.M., Assiri M.A., Almalki D.M., Alswat K. (2018). Diabetes-related distress assessment among type 2 diabetes patients. J. Diabetes Res..

[18] Hessler D.M., Fisher L., Polonsky W.H., Johnson N. (2016). Understanding the areas and correlates of diabetes-related distress in adults with type 1 diabetes. J. Diabetes Complicat..

[19] Chew B.H., Mohd-Sidik S., Shariff-Ghazali S. (2016). Diabetes-related distress, depression and distress-depression among adults with type 2 diabetes mellitus in Malaysia. PLoS ONE.

[20] Kamrul-Hasan A.B.M., Hannan M.A., Asaduzzaman M., Rahman M.M., Alam M.S., Amin M.N., Kabir M.R., Chanda P.K., Jannat N., Selim S. (2022). Prevalence and predictors of diabetes distress among adults with type 2 diabetes mellitus: A facility-based cross-sectional study of Bangladesh. BMC Endocr. Disord..

[21] Wang R.-H., Chen S.-Y., Lee C.-M., Lu C.-H., Hsu H.-C. (2023). Resilience, self-efficacy and diabetes distress on self-management behaviours in patients newly diagnosed with type 2 diabetes: A moderated mediation analysis. J. Adv. Nurs..

[22] AlSayah F., Yeung R.O., Johnson J.A. (2019). Association of depressive symptoms and diabetes distress with severe hypoglycemia in adults with type 2 diabetes. Can. J. Diabetes.

[23] Kamrul-Hasan A.B.M., Pappachan J.M., Nagendra L., Muthukuda D., Dutta D., Bhattacharya S., Shrestha D., Dhakal G.P., Sumanatilleke M., Raza S.A. (2025). Prevalence of diabetes distress among people with type 2 diabetes in South Asia: A systematic review and meta-analysis. World J. Diabetes.

[24] Schmitt A., Reimer A., Kulzer B., Haak T., Ehrmann D., Hermanns N. (2016). How to assess diabetes distress: Comparison of the Problem Areas in Diabetes Scale (PAID) and the Diabetes Distress Scale (DDS). Diabet. Med..

[25] Bubulac L., Dobjanschi C.G., Zivari M., Erena C., Tudor V., Albu C.-C. (2026). Psychological Distress and Health-Related Quality of Life in Romanian Adults with Diabetes Mellitus: A Cross-Sectional Study. Healthcare.

[26] Fisher L., Polonsky W.H., Hessler D. (2019). Addressing diabetes distress in clinical care: A practical guide. Diabet. Med..

[27] Snoek F.J., Bremmer M.A., Hermanns N. (2015). Constructs of depression and distress in diabetes: Time for an appraisal. Lancet Diabetes Endocrinol..

[28] Gahlan D., Rajput R., Gehlawat P., Gupta R. (2018). Prevalence and determinants of diabetes distress in patients of diabetes mellitus in a tertiary care centre. Diabetes Metab. Syndr. Clin. Res. Rev..

[29] Marciano L., Camerini A.L., Schulz P.J. (2019). The role of health literacy in diabetes knowledge, self-care, and glycemic control: A meta-analysis. J. Gen. Intern. Med..

[30] Butler A.M., Brown S.D., Carreon S.A., Smalls B.L., Terry A. (2022). Equity in psychosocial outcomes and care for racial and ethnic minorities and socioeconomically disadvantaged people with diabetes. Diabetes Spectr..

[31] Hessler D.M., Fisher L., Glasgow R.E., Strycker L.A., Dickinson L.M., Aréan P.A., Masharani U. (2014). Reductions in regimen distress are associated with improved management and glycemic control over time. Diabetes Care.

[32] Chew B.H., Vos R.C., Metzendorf M.I., Scholten R.J., Rutten G.E. (2017). Psychological interventions for diabetes-related distress in adults with type 2 diabetes mellitus. Cochrane Database Syst. Rev..

[33] Asshiddiqi M.I.N., Yodchai K., Taniwattananon P. (2021). Predictors of diabetes distress among older persons with type 2 diabetes mellitus in Indonesia. J. Res. Nurs..

[34] Albai O., Frandes M., Timar R., Timar B., Anghel T., Avram V.F., Sima A. (2021). The Mental Status in Patients with Diabetes Mellitus Admitted to a Diabetes Clinic After Presenting in the Emergency Room: The Application of the SCL-90 Scale. Diabetes Metab. Syndr. Obes..

